# Towards new TB vaccines

**DOI:** 10.1007/s00281-020-00794-0

**Published:** 2020-03-18

**Authors:** Benedict Brazier, Helen McShane

**Affiliations:** The Jenner Institute, Nuffield Department of Medicine, University of Oxford, Oxford, OX3 7DQ UK

**Keywords:** Tuberculosis, Vaccine, Immunity, Innate, Adaptive, BCG

## Abstract

*Mycobacterium tuberculosis* remains the leading cause of death attributed to a single infectious organism. Bacillus Calmette-Guerin (BCG), the standard vaccine against *M. tuberculosis*, is thought to prevent only 5% of all vaccine-preventable deaths due to tuberculosis, thus an alternative vaccine is required. One of the principal barriers to vaccine development against *M. tuberculosis* is the complexity of the immune response to infection, with uncertainty as to what constitutes an immunological correlate of protection. In this paper, we seek to give an overview of the immunology of *M. tuberculosis* infection, and by doing so, investigate possible targets of vaccine development. This encompasses the innate, adaptive, mucosal and humoral immune systems. Though MVA85A did not improve protection compared with BCG alone in a large-scale clinical trial, the correlates of protection this has revealed, in addition to promising results from candidate such as VPM1002, M72/ASO1E and H56:IC31 point to a brighter future in the field of TB vaccine development.

## Introduction

Consigned to history in the minds of many, tuberculosis (TB) is a disease far from defeated. The pathogen responsible, *Mycobacterium tuberculosis*, is the leading cause of death attributed to a single infectious organism [1]. Administered at birth as part of the Expanded Programme on Immunization (EPI) since 1974 [2], the vaccine Bacillus Calmette-Guerin (BCG) currently has 90% coverage globally. Despite this, one person dies of TB every 20 s [3]. The efficacy of BCG varies from 0 to 80% in protecting against pulmonary TB [4]. It is estimated that globally, BCG prevents only 5% of all vaccine-preventable deaths due to TB [5], the cruel irony being that BCG is least effective in the areas of the world where it is most needed. It is also these very areas where HIV infection rates are highest, a cohort for whom BCG is contraindicated, and for whom the risks of TB are higher. In areas where efficacy is preserved however, protection can be durable, with protective efficacy of over 50 years demonstrated in an Alaskan population [6].

Following the disappointing results from the first phase 2b efficacy trial with a new-generation subunit vaccine, MVA85A, the TB vaccine field had a period of review and reflection [7]. Even with the more promising results recently demonstrated with another subunit vaccine, M72/AS01E [8], it is clear that the understanding of immune correlates of protection was, and still is, insufficient. In this review, we have focussed on the current understanding of TB immunology, and how this knowledge can be utilised in the development of novel vaccines.

## BCG and efficacy

The derivation of BCG is a result of pathogenesis experiments carried out by Calmette and Guerin [9], who after 230 passages and 13 years declared the organism to be safe and protective against *M. tuberculosis* [10]. Major trials were initiated in the 1950s in the UK [11] and USA [12, 13], the results of which would set the tone for BCG trials of the future, in that the results were conflicting. The UK study involved 54,239 school children aged 14–15 years who were followed up for 20 years and demonstrated a protective efficacy of 77% [11]. In contrast, US trials involving 191,827 Puerto Rican school children aged 1–18 years [13] and 64,136 children aged above 5 years in Georgia and Alabama [12] showed a protective efficacy of only 16 and 29%, respectively, after 20 years follow-up. A critical moment in the acceptance of the variability in BCG efficacy came in 1980 with the publishing of the Chingleput trial [14]. Carried out in India and involving 73,459 individuals, in a highly surprising result, BCG provided no protection against TB [14]. Up to this point, much of the variance in reported efficacy had been put down to differences in trial design [15].

Though declared by Calmette to be a ‘virus fixe’ [16], multiple BCG strains have diverged [17]. Despite this, the impact of this divergence is insufficient to explain the variation in efficacy observed [18, 19]. The most significant factor influencing BCG efficacy that is supported by data is exposure to non-tuberculous mycobacteria (NTM) [19]. BCG efficacy correlates with latitude, as does NTM exposure, with BCG efficacy higher in temperate climates with lower NTM exposure [19]. NTM exposure is higher in tropical areas with lower BCG efficacy, such as Chingleput [20].

Although administering BCG vaccination shortly after birth reduces the risk of prior NTM exposure, such timing is frequently not possible in resource poor settings, particularly rural ones, and in areas where HIV infection needs to be excluded prior to BCG administration. A study from The Gambia has previously demonstrated NTM exposure in BCG-naive 4-month-old infants [21]. A study in mice has demonstrated that exposure to NTM post-BCG vaccination can reduce the protective efficacy of BCG [22].

There are two hypotheses proposed for the mechanism by which NTM exposure reduces BCG efficacy: masking and blocking [23, 24]. The masking hypothesis states that background immunity generated by prior exposure to NTM means that any added benefit of subsequent BCG vaccination is minimal [23]. The blocking hypothesis suggests that the pre-existing immune response to antigens shared by NTM and *M. tuberculosis* results in rapid eradication of BCG, such that the amount of available BCG derived antigens is limited, diminishing immunity [24]. Whilst these two hypotheses are not mutually exclusive, mathematical analysis of the BCG-REVAC trial suggests that blocking is the main mechanism [25].

One implication of this for the development of novel vaccines is that a dominant blocking mechanism suggests that a new vaccine need only be as good as BCG to have measurable effect, as long as it is not blocked by prior sensitisation [26]. Importantly, pre-exposure to NTM does not seem to affect the efficacy of non-replicating subunit vaccines [24].

## The early innate response to *M. tuberculosis* exposure

The role of an early innate immune response in preventing or clearing early *M. tuberculosis* infection is increasingly recognised [27]. A subset of people exposed to *M. tuberculosis* are capable of achieving sterilising immunity post-exposure, termed early clearance [28]. The presence of latent *M. tuberculosis* infection (LTBI) is usually assessed via a tuberculin skin test (TST) or interferon-gamma release assay (IGRA), which when positive indicates immune sensitisation to *M. tuberculosis*, i.e. that an infection has occurred. In studies with a minimum of 2 years of longitudinal observation, the frequency of early clearance in household contacts of TB patients ranged from 3.4 to 26.8% when using TST conversion [28–30], whilst another study utilising IGRA conversion suggested 58% clearance [28]. A number of immune mechanisms for this have been proposed, including innate immune responses [31], antibody-innate cell interaction via Fc receptors [32] and lung resident T cells [33]. Investigations into a genetic basis for early clearance have found single nucleotide polymorphisms in NOD, and NRAMP1, suggesting a role for innate immunity in this process [34]. NOD2 signalling is already known to be increased post-BCG by up to a year through trained immunity [35].

The different components of an innate immune response to *M. tuberculosis* exposure are outlined below. Whilst vaccine development has traditionally focussed on the induction of an adaptive immune response, adjuvants that modulate innate immune pathways and a vaccine delivered by aerosol to the respiratory mucosa might target these pathways [36].

## Airway epithelial cells

*M. tuberculosis* enters the body via small aerosolised droplets, inhaled into the airways [37, 38]. Though alveolar macrophages (AMs) are the principal target of these bacilli, they are also capable of infecting human lung epithelial cells [39]. Airway epithelial cells (AECs) express a variety of pattern recognition receptors (PRRs) in addition to surfactant proteins that bind components of the mycobacterial cell wall [40]. This epithelial recognition of *M. tuberculosis* activates a number of signalling pathways, inducing the production of cytokines such as tissue necrosis factor α (TNFα) and interferon-ɣ (IFN-ɣ), and chemokines such as IL-6 and IL-8 [41–44]. Furthermore, AECs are potent responders to cytokines such as IL-1β and type 1 interferons released by infected macrophages, enabling efficient cross-talk [31]. AECs are even capable of directly presenting intracellular antigens to resident CD8+ T cells via MHC class I molecules, stimulating IFN-ɣ production [45].

### Alveolar macrophages

AMsare some of the first cells of the immune system to come into contact with *M. tuberculosis*, phagocytosing the bacilli. This phagocytosis is mediated principally by complement receptor 4 (CR4) [46]; however, AMs are highly heterogeneous in their phagocytic potential, with only 20% of AMs in culture becoming infected by *M. tuberculosis* even with high bacterial loads [47]. *M. tuberculosis* infection induces a phenotypic shift from oxidative phosphorylation (M2, anti-inflammatory) to aerobic glycolysis (M1, pro-inflammatory), resulting in increased IL-1β levels and decreased IL-10 levels [48]. This polarisation to an M1 phenotype aids antimicrobial activity [49]. However, the TNF produced by AMs may be counterproductive, with exogenous application of TNF increasing both intracellular bacterial load and the number of infected AMs [47, 49].

In a mouse model, the depletion of macrophages prior to a lethal infection with *M. tuberculosis* improved survival [50], yet specifically depleting activated macrophages was detrimental [51]. The protective effect of AMs may be dependent on their subtype.

### Neutrophils

Another cell type implicated in the initial response to *M. tuberculosis* exposure are neutrophils, among the first immune cells to migrate to the site of infection [52]. Neutrophils secrete antimicrobial enzymes such as α-defensins and lactoferin [53], chemokines such as IP-10 [54] and MCP-1 [55] and cytokines such as TNFα [56]. Neutrophils kill *M. tuberculosis* primarily through the respiratory burst and phagocytosis [57]. Whilst this response may appear to be beneficial, the reality is more complex, as is exemplified in the case of lipocalin-2. Lipocalin-2 is a constituent of neutrophil secondary granules, blocking bacterial scavenging of iron [58]. In mice, lipocalin-2 increases susceptibility to *M. tuberculosis* prior to granuloma formation [59], potentially via increasing the amount of iron available to intracellular mycobacteria [59].

In humans, peripheral neutrophilia is a hallmark of TB disease and is a poor predictor of outcome [60], with neutrophil depletion decreasing *M. tuberculosis* killing [57]. A neutrophil-driven interferon inducible gene profile consisting of both IFN-ɣ and IFN-αβ was one of the principal components of an 86 transcript signature of active TB [61]. As a predominant cell type infected by *M. tuberculosis*, the evidence suggests a role for neutrophils in the pathogenesis of TB, a possible granulocytic Trojan horse [62].

### Other innate cells

There are many other innate cell types for which there is some evidence for a role in protection against mycobacterial infection, including NK cells [63, 64], ɣδ T cells [65–67] and mucosal-associated invariant T cells [68, 69]. Innate lymphoid cells (ILCs) share features of both the innate and adaptive systems, and are categorised into three subsets [70]. Group 3 ILCs (ILC3s) mediate early protective immunity against *M. tuberculosis*, recruited via a CXCL13-CXCR5 axis to inducible bronchus-associated lymphoid tissue (iBALT)-associated granulomas [71].

iBALT, like other lymphoid organs, are composed of segregated T and B cell areas [72]. These highly organised structures form spontaneously in response to pulmonary infection [73]. iBALT surrounds the granulomas in *M. tuberculosis* infected humans [74], NHPs [75] and mice [76]. The absence of iBALT is associated with active disease, whereas presence is associated with containment of infection and maintenance of latency [74, 77].

**Fig. 1:**
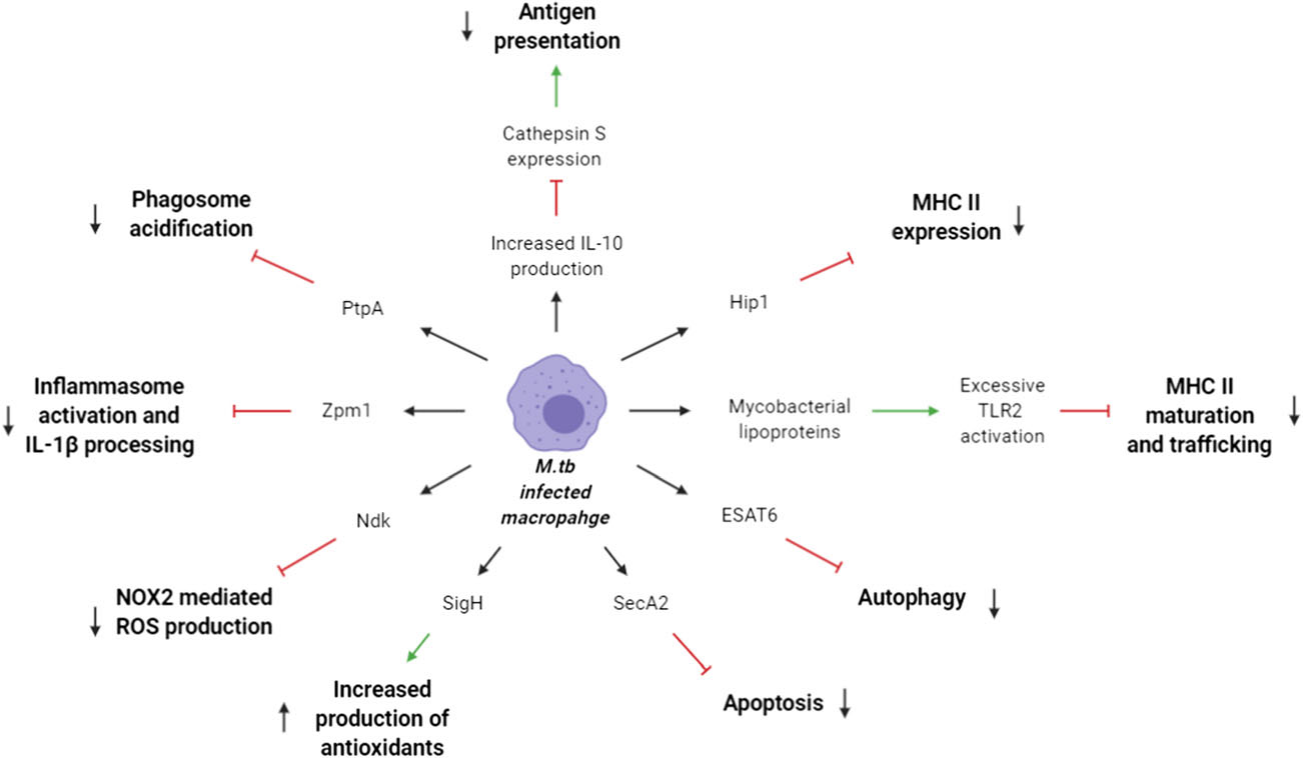
*M. tuberculosis* utilises a variety of means to undermine the ability of an infected macrophage to destroy the mycobacilli, thus also avoiding the presentation of *M. tuberculosis* antigens to the adaptive immune system

ILC3 produce IL-17 and IL-22 in response to IL-23 stimulation [78], the IL-23 produced by *M. tuberculosis* infected lung cells [71]. Early neutralisation of IL-23 in mice increased early *M. tuberculosis* burden, resulting in a decreased formation of iBALT, whilst mice lacking ILC3 exhibit a reduction in the accumulation of early alveolar macrophages [71]. CXCL13 is induced in the lungs during *M. tuberculosis* infection, recruiting lymphocytes through CXCR5 to mediate their spatial organisation within iBALT [77]. IL-17 is one of the key mediators of increased CXCL13 levels [79] and will be covered later in the section on mucosal immunity. An overview of this axis can be seen in Fig. 1.

Using a mycobacterial growth inhibition assay, a new subset of innate cell has been found to be strongly associated with trained innate immunity [80]. Control of mycobacterial growth was associated with the presence of a non-classical CD14-dim monocyte population [80]. These cells are highly motile and able to release multiple cytokines, yet are weakly phagocytic [81]. One of the chemokines secreted includes CXCL10 (a CXCR3 ligand), production of which correlating strongly with BCG growth reduction [80]. Importantly, CXCR3 ligands such as CXCL10 are associated with trained immunity [80].

### Trained immunity

Trained immunity describes epigenetic changes to the genes of the innate immune system, resulting in a memory like function. BCG vaccination has been shown to alter the acetylation and methylation of innate immune genes, amplifying the response to subsequent stimulus [82]. This may be the basis of the reported non-specific protective effect of BCG, which in some studies has been suggested to reduce mortality in the first 6–12 months of life [83]. This trained immunity induced by BCG can confer heterologous protection against other pathogens in vitro [35]. BCG induced a two-fold increase in monocyte derived cytokines such as IL-1β and TNFα in response to an in vitro bacterial and fungal challenge. Whilst for the most part this is a fairly short lived response, intravenous (IV) BCG has been shown to drive epigenetic changes in haematopoietic stem cells (HSCs), thus potentially impacting immunity over the longer term [84].

## Harnessing the innate immune system

### Targeting dendritic cells and haematopoietic stem cells

Activation of the adaptive immune response requires the presentation of *M. tuberculosis* antigens to T cells by dendritic cells and macrophages. There is a significant delay between infection and the induction of an adaptive immune response, which in humans is first detectable 18–20 days after infection [85]. Migration of *M. tuberculosis* to the draining lymph nodes only occurs 7–9 days post-infection; this delay allows for a marked expansion in bacterial population [85].

Methods of activating dendritic cells (DCs) via adjuvants may improve the protective efficacy of vaccines. Amphiphilic-CpG (amph-CpG), a modified TLR9 agonist in mice, enhances the T cell response to peptide vaccination in addition to upregulating CD103 [86]. When used in combination with Fgk4.5, an agonistic CD40 antibody, DCs are activated resulting in early *M. tuberculosis* control [87]. The use of TLR ligands requires care however, with the administration of polyl:C (a TLR3 ligand) exacerbating inflammation and increasing *M. tuberculosis* burden [88].

Intravenous BCG vaccination is capable of priming HSCs, resulting in a polarisation towards the myeloid lineage, correlating with an upregulation of IFN-ɣ [84]. This long lasting effect of IV BCG on the innate immune system may explain the superior protection seen in NHPs using this route of vaccination [89, 90]. Whilst this work provides important proof of concept for immune mechanistic work, such a route is unlikely to be a feasible approach for neonates or in low resource settings.

### Targeting macrophages

**Fig. 2:**
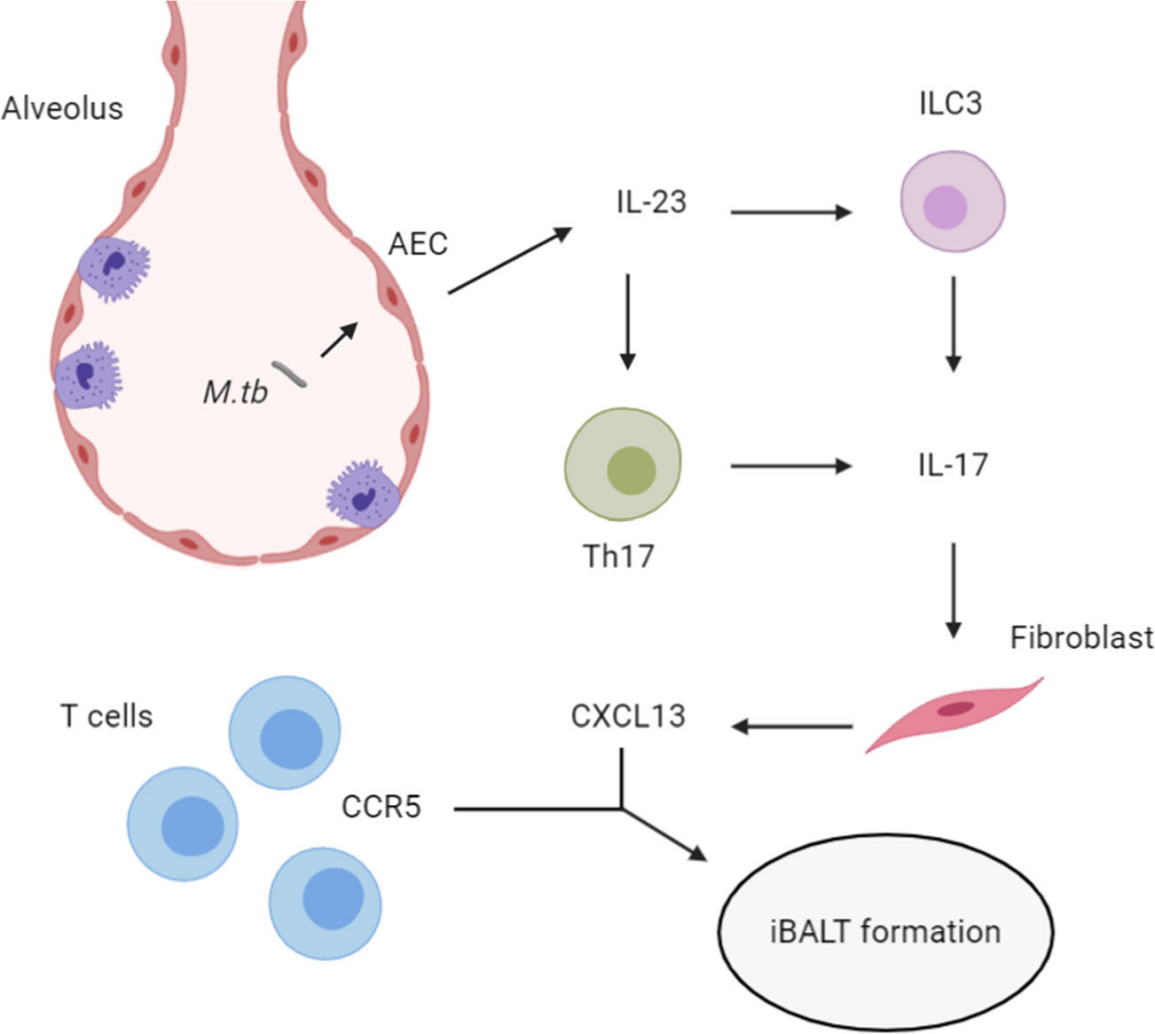
*M. tuberculosis* infection of AECs results in increased IL-23 production, which increases IL-17 release from ILC3 and Th17 cells, in turn increasing CXCL13 production by fibroblasts. This helps attract CCR5+ T cells, helping to form iBALT

*M. tuberculosis* has a variety of means to mitigate its destruction by macrophages, enabling it to survive in macrophages without destruction. An overview of this can be seen above in Fig. 2 [91–102]. The immune evasion and immunosuppression of macrophages by *M. tuberculosis* result in less effective antigen presentation, resulting in a less-effective adaptive immune response [103]. Increasing the rate of apoptosis would result in the release of apoptotic vesicles carrying *M. tuberculosis* to uninfected DCs, thus allowing antigen presentation and activating adaptive immunity [104].

*M. tuberculosis* inhibits inflammasome activation and IL-1β processing through zinc metalloproteinase 1 (zmp1) [91]. A recombinant BCG vaccine candidate in late preclinical development, BCG ∆zmp1, demonstrates increased phagosome maturation and phagolysosmal fusion, aiding antigen presentation resulting in improved protection [105–107].

Negating the capacity of *M. tuberculosis* to inhibit and neutralise reactive oxygen species (ROS) production by macrophages is yet another target for vaccine design. In doing so, macrophages would be better able to present antigens, thus improving immunogenicity and efficacy [108–111]. SigH is among these potential targets, involved in orchestrating an *M. tuberculosis* stress-response pathway [92]. A duplication of SigH in a majority of BCG strains further reduces the susceptibility of BCG to ROS [112]. The vaccine candidate *M. tuberculosis*∆sigH shows greater levels of apoptosis and a markedly stronger innate immune response [113], with sterile protection observed in some TB lesions in preclinical studies [113].

In addition to inhibiting phagolysosomal maturation/fusion and ROS inhibition, *M. tuberculosis* is also capable of inhibiting the maturation and trafficking of MHC molecules [114]. The activation of TLR2 by mycobacterial lipoproteins induces excessive signalling, resulting in an inhibition of MHC II expression [93–95]. A reduction in cathepsin S (Cat-S) expression is seen in *M. tuberculosis* infected macrophages [96], associated with the induction of IL-10 [97]. Addition of anti-IL-10 antibodies has been shown to restore macrophage Cat S expression, increasing antigen presentation [97]. BCG-CatS is a recombinant BCG vaccine engineered to secrete active Cat S, and stimulates much stronger macrophage presentation of Ag85B to CD4+ T cells than BCG [115].

Another strategy may be to target the autophagy and apoptosis pathways. *M. tuberculosis* inhibits autophagy through ESAT6, secreted by the ESX-1 secretion system [98]. As this is encoded in RD1 (region of difference 1), which is absent in BCG [116], autophagosome maturation is not inhibited with BCG [117]. A recombinant strain of BCG expressing ESX1 (BCG::ESX-1) is more protective than wild type BCG in mouse and guinea pig models, although this recombinant strain is more virulent [118]. By utilising the evolutionarily more distant ESX-1 from *M.marinum*, BCG::ESX-1^Mmar^ has comparable efficacy and immunogenicity to BCG::ESX-1*M. tuberculosis*, yet has a safety profile comparable with BCG Pasteur in preclinical models [119].

### VPM1002

The most clinically advanced recombinant BCG (rBCG) strain in development is rBCG ∆ureC::Hly, also known as VPM1002. This construct replaces the urease C gene with that of listeriolysin O, a haemolytic (hly) pore forming protein originating from *Listeria monocytogenes* [120, 121]. Listeriolysin O forms transmembrane β-barrel pores in the phagolysosome membrane, thus allowing the escape of antigens and mycobacterial DNA into the cytosol [122, 123]. By replacing urease C, the BCG construct is less able to alkalinise the phagolysosome, ensuring the activation of listeriolyin, which is active at a pH of 5.5 [124]. The net effect is designed to increase the levels of apoptosis, autophagy and inflammasome activation [125].

In mice, VPM1002 was cleared faster than BCG [126] and was also safer in immunodeficient SCID mice [121]. In both guinea pigs and non-human primates, the safety profile of VPM1002 has been found to be comparable with that seen with BCG [127, 128]. Grode et al. found VPM1002 to have greater protective efficacy compared with BCG in BALB/c mice [121]. Phases I and IIa studies found VPM1002 to be safe and capable of eliciting a strong immune response, at least comparable with BCG [129, 130].

A phase IIb trial in South Africa, evaluating the safety and immunogenicity of VPM1002 in comparison with BCG in both HIV unexposed and HIV-exposed uninfected (HEU) BCG naïve newborns (NCT02391415), has now concluded with data awaiting public release. A phase III trial is also underway in India (NCT03152903), investigating efficacy against relapse in adolescents and adults who have been recently treated for active TB.

## The importance of a Th1 adaptive immune response

IFN-ɣ, the hallmark cytokine of a pro-inflammatory Th1 response, is critical for protection against *M. tuberculosis* [131, 132]. Individuals who are CD4+ T cell deficient, such as those infected with HIV, or those with inborn genetic errors of IFN-ɣ signalling are highly susceptible to *M. tuberculosis*, thus indicating the importance of the Th1 response [133–135]. Whilst essential to controlling *M. tuberculosis* infection, IFN-ɣ may not be sufficient [136]. Deficiency in other factors such as IL-1, IL-6 and TNFα is also important for protection in murine and human studies [136].

Most studies look at peripheral, systemic immune responses, but there is increasing interest in the lung environment. The pulmonary CD4+ T cell response can be divided into two subsets, one in the lung parenchyma, and one residing within the vasculature [137]. The parenchymal effectors are PD-1^hi^/CD69^hi^ CD4+ T cells, which are highly proliferative, in contrast with the more terminally differentiated KLRG1^hi^/T-bet^hi^ CD4+ cells resident in the vasculature [137–141]. These KLRG1^hi^ cells produce more IFN-ɣ [140] and are the most abundant subset in the lung at the peak of clonal expansion [137]; however, they are very poor at entering the lung parenchyma [142].

## IL-17

There is also evidence that a Th17 response may be implicated in protection. IL-17 is produced principally by Th17 cells, which require both TGF-β and IL-16 for initiation [143–145], in addition to IL-23 in order to become an established population. These IL-17-producing cells provide a surveillance function in the periphery [146], though in excess they can are associated with excess neutrophil recruitment [147, 148] and autoimmune disease [146, 149, 150]. Pathogenic overproduction of IL-17 is restricted by IFN-ɣ [151–153], limiting neutrophil accumulation and coincident lung inflammation during *M. tuberculosis* infection. IL-17 itself drives Th1 response by overcoming IL-10 inhibition [154], thus IL-17 and IFN-ɣ have significant interplay [155]. Th17 cells have been found within the pulmonary lesions of TB patients, in addition to the less well characterised Th1/Th17 cells [156, 157]. Also known as Th1* cells, these are capable of producing both IFN-ɣ and IL-17, but their role in TB is still unclear [156].

In addition to their role in granuloma formation, there also appears to be a link between IL-17 and protective antibodies. Using the TB susceptible DBA/2 mouse strain, it was found that intranasal but not subcutaneously administered BCG conferred robust protection against pulmonary TB [158]. This was associated with an IL-17-based *M. tuberculosis*-specific mucosal immune response following intranasal vaccination [158]. Neutralisation of IL-17 in vivo abrogated the *M. tuberculosis*-specific IgA secretion seen in the respiratory airways and reduced lung expression of polymeric immunoglobulin receptor (pIgR), which translocates IgA into the airway lumen [159].

## Mucosal immunity

There is increasing evidence from animal models that delivery of a vaccine direct to the respiratory mucosa may be a more protective route of vaccination. An understanding of the different T cell subsets within the lung would inform the design of vaccines targeting this route.

### Lung T cell subsets

Thus far, three main subsets of lung resident memory cells have been defined: T effector memory cells (T_EM_), T central memory cells (T_CM_), and T resident memory cells (T_RM_). Much of the available data on this has come from murine studies. It is not always clear how these findings relate to NHPs and humans. Most of the lung resident T memory cells are of the T_EM_ phenotype [160], CD44^hi^ CD62L^lo^ CD127^hi^ [141, 161]. The T_EM_ subset act in the first line of defence [162], predominantly secreting Th1 cytokines [160]. They are able to recirculate between blood, non-lymphoid tissues and lymph [163]. Studies of individuals with LTBI have demonstrated an increased level of the exhaustion marker PD-1 on T cells, perhaps due to continuous antigenic stimulation [164]. T cells from BCG vaccinated individuals were CD27+ but had low PD-1 expression, indicating an earlier stage of differentiation [164]. Despite this, the antigen-specific CD4+ T cell response of BCG-vaccinated human new-borns wanes over the first year of life, suggesting that the T_EM_ population induced is unable to maintain persistent memory [165]. In response to continuous antigen exposure, T_EM_ become terminally differentiated T effector (T_eff_) cells, losing the ability to proliferate and migrate into the lung parenchyma, expressing the KLRG1 marker [166, 167].

In contrast, IL-2-producing T_CM_ have a high proliferative capacity [168], usually CD62^hi^ CD127^hi^ [161], and derive from KLRG1^−^ precursors [169]. This cell population is capable of rapid proliferation, evolving into large numbers of pro-inflammatory effectors upon antigen re-exposure [168]. The lack of T_CM_ induction by intradermal BCG may underlie the loss of protective efficacy with time [170], supported by findings that prevention of T_CM_ exiting the lymph nodes has no influence on the protection provided by BCG [171]. This indicates that BCG promotes mainly T_EM_- and T_eff_-based responses [171].

A recent study has challenged the conventional view that T_CM_ are necessary for vaccine-induced protection. A recombinant CMV-vectored TB vaccine achieved very high levels of protection against *M. tuberculosis* challenge in NHPs which was associated with the induction of T_EM_ and transitional effector memory T cells (T_TrEM_), not T_CM_ [172]. The ability of T_CM_ to confer greater protection than T_EM_ is possibly best shown by adoptive transfer of the separate T subsets (Kaufmann et al.), in which T_CM_ markedly protected against TB in contrast with T_EM_ and T follicular helper (T_FH_) cells [126]. These T_CM_ cells had characteristic CXCR5^+^ CCR7^+^ expression and CXCR5 expression correlating with decreased lung pathology [126].

In mice, VPM1002 delivered subcutaneously induced a significantly increased T_CM_ response compared with BCG, which was associated with improved protection after *M. tuberculosis* challenge [126]. Adoptive transfer of T_CM_ specific for *M. tuberculosis* conferred protection, whereas adoptive transfer of T_FH_ alone did not [126].

T_RM_ are CD44^hi^ CD62L^lo^ CD69^+^ CD103^+^ in phenotype [141], like T_CM_ deriving from KLRG1^−^ precursors [169]. T_RM_ permanently reside in non-lymphoid tissue, making them strategically placed for a rapid recall response [138]. As a group, T_RM_ are highly heterogeneous, with some CD4+ T_RM_ displaying a regulatory profile (Foxp3^hi^ IL-10^hi^) and others with a more effector profile (T-bet^+^) [141]. In contrast, airway resident CD8+ T_RM_ cells are more homogenous, expressing predominantly Th1 cytokines [141]. In addition to their cytolytic role, CD8+ T_RM_ are also capable of activating bystander NK and B cells through IFN-ɣ, TNFα and IL-2 [173]. Maintenance of T_RM_ may be reliant on the presence of live bacilli, as clearance of BCG in mice with chemotherapy abrogates the antigen-specific CD4+ T cell response [166]. Of all the T memory cell subtypes, the mucosal transfer of CD8+ T_RM_ cells was associated with the most protection against *M. tuberculosis* challenge on a per-cell basis [141].

Despite promoting lung-localised T_RM_, mucosal boosting with a protein/adjuvant candidate vaccine, H56:CAF01, did not enhance protection [174]. H56 is a subunit vaccine, a fusion protein of the *M. tuberculosis* antigens Ag85B, ESAT-6 and Rv2660c [175], which has been combined with the liposome adjuvant CAF01. The parenteral priming followed by mucosal boosting did enhance early lung T cell response; however, mucosal boosting did not alter the cytokine profile nor conferred added protection [174]. H56:IC31 administered systemically has been evaluated in a phase 2a trial (NCT01865487) [176] and is currently recruiting for another larger scale phase 2 trial (NCT03512249).

In summary, less-differentiated CD4+ T cells seem to provide greater protection than more-differentiated effector T cells. Vaccine strategies should therefore attempt to induce these cell populations, which appear to be related to dose and persistence of the vaccine construct [165, 177].

### Mucosal TB vaccines

The concept of delivering a TB vaccine direct to the respiratory mucosa is nothing new. Nebulised BCG was demonstrated to be safe and immunogenic in terms of tuberculin skin test conversion in 1968 [178]. There are concerns about intransal delivery after transient cases of facial nerve palsy following nasal subunit vaccination in two phase 1 clinical trials [179, 180]. Furthermore, there were worries that a post-exposure vaccine could trigger Koch’s phenomenon, in which reinfection is marked by rapidly developing necrotic lesions caused by hypersensitivity to the mycobacteria [181]. To date, this concerns appear unfounded, at least in BCG-primed individuals [182].

Aerosolised MVA85A, a modified Vaccinia virus Ankara expressing Ag85A, was evaluated in a proof-of-concept phase 1 trial (NCT01497769) in BCG vaccinated healthy adults [182]. In this trial, respiratory adverse events post-aerosol were rare, with no difference in occurrence compared with placebo [182]. Aerosol delivery induced more potent brochoalveolar lavage Th1 responses compared with intradermal vaccination and comparable systemic responses [182].

Adenoviruses are another promising candidate for use in a mucosal TB vaccine due to their natural tropism for respiratory epithelium [183]. Two adenovirus-based TB vaccines are AdHu5Ag85A, which has demonstrated T cell responses despite pre-existing adenoviral immunity [184], and ChAdOx1.85A [185]. Both are currently being evaluated as a nebulised vaccine (NCT02337270 and NCT04121494). An adenovirus AdHu35 expressing the *M. tuberculosis* antigens Ag85A, Ag5B and TB10.4, AERAS-402, had demonstrated robust cellular immune responses in the lungs of rhesus macaques, however this failed to confer added protection [186]. Whilst in mice the accumulation and retention of memory CD4+ and CD8+ T cells within the airway lumen correlated with protection against TB, this was not observed in the macaques. This was potentially due to the very large doses of *M. tuberculosis* used in the macaque trial [186].

Mucosal BCG vaccination has been shown to confer superior protection in the lungs compared with intradermal BCG in mice [139], and parenteral administration in guinea pigs [187] and macaques [188], associated with greater numbers of T_RM_ and an enhanced proliferative capacity of lung parenchymal CD4+ T cells [139, 141]. The superior protection was specific to the lungs, with protection in the spleen equal to that conferred by the intradermal route [139]. CXCR3 expression, key to the recruitment of CD8+ T cells [189], was only found in lung parenchymal CD4+ T cells with mucosal BCG vaccination [139]. A recent study in NHPs has demonstrated a superior protective effect of mucosal BCG immunisation compared with intradermal immunisation against low-dose repeated *M. tuberculosis* infection [190]. The mucosally immunised group showed higher local levels of polyfunctional Th17 cells, IL10 and mucosal IgA [190]. Though this work is highly promising, novel methods for the immunomonitoring of aerosol vaccination are necessary, due to the invasive nature of bronchoscopy and bronchoalveolar lavage. Induced sputum is one possibility, having been used before as an immunoassay in TB patients [191, 192].

## Humoral immunity

The role of humoral immunity in protection against TB was for a long time discounted. When cynomolgus macaques were treated with rituximab, a B cell depleting agent (but not plasma cell depleting), the overall disease progression and outcome of *M. tuberculosis* infection in the acute phase was unaltered [193]. Though no change in outcome was seen, there was a significant increase in IL-2, IL-10 and IL-17 producing T cells within rituximab treated macaques, though IL-6 and IL-10 levels were lower in the granulomas themselves [193]. IL-6 and IL-10 are both secreted by B cells [194, 195], IL-6 increasing T cell development [196] and B cell expansion [194, 197]. In B cell-deficient mice, BCG was less effective as a result of the dysregulated IL-17 production [198, 199], the elevated IL-17 resulting in greater levels of phagocytosis of BCG by neutrophils rather than monocytes [200].

By comparing the antibody profiles between those with active TB and LTBI, a functional role for antibodies is emerging. An unbiased systems serology approach found nine specific antibodies capable of distinguishing the two groups, LTBI or active TB [32]. Latent infection is associated with unique antibody glycosylation and Fc functional profiles, which drives innate immunity to kill intracellular *M. tuberculosis* [32]. Fc glycosylation could be a potential future biomarker, with the differences reflecting differential B cell priming [201]. Furthermore, patients with LTBI show superior NK cell mediated cytotoxicity, associated with increased levels of binding to FcɣRIII, driving NK cell activation [32, 202]. Whether these differences are a pathological mechanism of *M. tuberculosis* persistence or outcome of successful control are thus far unclear.

Further clues as to a role for humoral immunity come from health care workers (HCWs) with occupational exposure to *M. tuberculosis* [203, 204]. HCWs have slightly higher titers of *M. tuberculosis*-specific IgA than those with active TB, with 7/12 isolated IgA mAbs capable of restricting *M. tuberculosis* growth compared with 0/16 IgG mAbs [203]. In another study, no patients with active TB made protective antibody responses, whereas a subset of patients with LTBI and HCWs had antibodies capable of restricting *M. tuberculosis* growth [204]. This growth restriction was completely negated by the absence of CD4+ T cells, perhaps because of a requirement for immune complexes [204].

The protection shown by IgA but not IgG points to the importance of invariant antibody function in protection. *M. tuberculosis* infection in mice lacking activating FcɣR ɣ-chain results in more severe immunopathology during disease due to higher IL-10 levels, further supporting the importance of Ab-Fc function [205]. FcRs can be activating or inhibitory, with heterogeneity between individuals impacting whether B cells have a pro or anti-inflammatory impact at the level of the granuloma [205]. It is possible that opsonising antibodies better enable *M. tuberculosis* to be internalised via phagocytosis into target macrophages [203].

Though the natural protective effect of antibodies appears small, this does not necessarily mean that a humoral vaccine would fail, only that different antigens need be found. In the laboratory, *M. tuberculosis* is frequently grown in detergent, stripping the capsule [206]; these capsular antigens have been shown to generate IFN-ɣ and T cell responses in addition to high titers of antibody [207]. Carbohydrate-protein conjugate vaccines against arabinomannan in addition to a peptide mimotope against LAM have demonstrated efficacy in murine models [208, 209].

## Correlates of protection

The lack of validated immune correlates of protection is one of the greatest challenges in TB vaccine development. Identification and validation of such correlates is possible only when samples from successful placebo controlled efficacy trials become available, requiring a comparison of the immune responses in vaccinated and unvaccinated individuals protected against *M. tuberculosis* in addition to those not protected [210].

The greatest potential for immune insight would be if the leading candidate TB vaccines induced a diverse immune response. However, in a recent comparison of antigen-specific T cell responses from human clinical trials, the functional profiles suggested a lack of response diversity, with the main difference in the magnitude of response [211]. This comparison involved AERAS-402, H1:IC31, M72/ASO1E, ID93+GLA-SE, H56:IC31 and MVA85A [211].

## The state of the pipeline

### Whole cell vaccines

Due to the difficulties in identifying antigens capable of generating a protective response, whole cell derived vaccines have gained increasing interest [212]. Whilst advantages with whole cell vaccines include a comprehensive antigen repertoire and similarity to natural infection [213], there are worries that this may simply induce a similarly semi-effectual immune response to that seen with natural *M. tuberculosis* infection [212].

Results reported by Nemes et al. have raised interest in the use of BCG re-vaccination, rather than simply focussing on novel vaccines [214]. This is due to a finding of 45.4% efficacy of BCG revaccination against sustained QuantiFERON TB-GOLD (QFT) conversion [214]. This was a phase IIb prevention of infection (POI) trial of H4:IC31 vs. BCG revaccination in an adolescent cohort [214]. H4 is a subunit vaccine consisting of Ag85B and TB10.4, which do not cross-react with QFT, combined with the IC31 adjuvant [214]. Though H4:IC31 induced significant increases in Ag85B and TB10.4-specific CD4+ T cell responses, neither H4:IC31 nor BCG re-vaccination prevented initial QFT conversion, failing to meet the primary endpoint [214].

Recombinant BCG strategies can broadly be divided into two camps, the first of which being those that overexpress *M. tuberculosis* immunodominant antigens such as rBCG30. rBCG30 overexpresses Ag85B [215] and was shown to be well tolerated and more immunogenic than BCG in a phase 1 trial [216]. The second strategy involves the modification of BCG for more effective antigen presentation and T_CM_ induction. Some examples of BCG-based vaccines have been described throughout this review, whether they work through a return of lost virulence factors as in the case of BCG::ESX-1 [118], aiding apoptosis as achieved by BCG::BAX [217] or through aiding phagolysosome escape as seen with VPM1002 [120].

The clinical development of one recombinant BCG strain, AERAS-422, an rBCG overexpressing three mycobacterial antigens and expressing perfringolysin, was terminated after 2/8 immunised healthy volunteers developed shingles after the reactivation of varicella zoster virus [218].

Rather than creating a more immunogenic/virulent BCG, another tactic is to attenuate *M. tuberculosis* itself. MTBVAC has deletion of the transcription factor phoP [219], which would otherwise control intracellular adaptation of the mycobacteria and promote ESAT-6 secretion [220, 221], and deletion of fadD26, required for synthesis of virulence associated cell wall lipids (pthiocerol dimycocerosates) [222, 223]. A phase 2 trial of MTBVAC vs BCG in adults and neonates has just reported (NCT02729571), finding it to be safe and immunogenic, paving the way for larger scale trials [224].

Heat-inactivated *Mycobacterium vaccae* has been approved in China, yet there is little publicly available information from the Chinese trials [225], with the DarDar trial the only trial clearly showing clinical efficacy, although the primary outcome was not reached [226]. Now re-branded DAR-901, grown in broth instead of agar [212], it is currently being evaluated in a phase 2b trial (NCT02712424). *Mycobacterium indicus pranii* (MIP) is a non-pathogenic mycobacterium, FDA approved as a leprosy vaccine [227]. MIP has been shown to be safe in pulmonary TB patients undergoing retreatment for TB [228].

RUTI, detoxified and fragmented *M. tuberculosis* within liposomes, is an immunotherapeutic agent to reduce the extent and duration of required drug treatment of active TB [229] and is currently being evaluated in a phase 2a trial (NCT02711735).

### Subunit vaccines

One means of retaining the protective effect of BCG is using a prime-boost strategy, in which BCG is still used, but with the addition of a heterologous vaccine booster [230]. Subunit vaccines require identification of protective antigens and also identification of an appropriate antigen delivery system which is usually a protein/adjuvant combination or a recombinant viral vector [231]. Subunit-based vaccines allow the triggering of immune memory without the safety concerns of a live vaccine, in addition to giving the short exposure that most favours T_CM_ formation [166, 232].

The final analysis of the post-exposure phase IIb trial of the subunit vaccine M72/ASO1E has shown 50% efficacy against progression to TB relative to placebo in patients already latently infected with *M. tuberculosis* [233]. M72 is a fusion protein derived from the antigens *M. tuberculosis*32A and *M. tuberculosis*39A, the adjuvant also a component of the malaria vaccine RTS, S/AS01 [233]. This was an extremely important result, demonstrating the potential of novel TB vaccines in pre-sensitised populations. One key important question arising from these data are whether this vaccine would confer protection in *M. tuberculosis*-uninfected subjects. If not, the efficacy will be lower in areas of the world where *M. tuberculosis* infection prevalence is lower. Preclinical studies demonstrate some level of protection in *M. tuberculosis*-uninfected NHPs and guinea pigs [234, 235].

The main candidate subunit vaccines are M72/ASO1E [233] and H56:IC31 [176], previously mentioned, in addition to ID93:GLA-SE [236]. The latter is a fusion of four *M. tuberculosis* antigens (Rv1813, Rv2608, Rv3619 and Rv3620) [236] combined with the TLR-4 agonist adjuvant GLA-SE [237], which has completed a phase 2a trial successfully [238].

### Viral-based vaccines

With their natural tropism, viral vectors allow greater targeting than subunit vaccines. The benefits of adenovirus-based vaccines have previously been discussed. TB/FLU-04l uses another respiratory epithelium tropic virus, the influenza H1N1 as a base, expressing the Ag85A and ESAT-6 antigens [227].

MVA85 was the first TB vaccine to enter efficacy trials since 1968 [7]. MVA (Modified Vaccinia Ankara) is an attenuated strain of Vaccinia virus, unable to replicate, with the addition of Ag85A [239]. Despite promoting powerful Th1 responses in early clinical trials [240], MVA85A failed to demonstrate protection in a preventative pre-exposure phase IIb trial in BCG-vaccinated infants [7].

Despite this failure, trials involving MVA85A have identified potential immune correlates [241]. In BCG-vaccinated infants, activated HLA-DR^+^ CD4+ T cells were associated with an increased risk of TB, a result confirmed in an adolescent cohort [241]. A linear effect was also seen with higher numbers of IFN-ɣ secreting BCG-specific T cells associated with a greater reduction in the risk of TB disease, in addition to Ag85A-specific IgG correlating with non-progression to disease [241]. This illustrates the importance of storing immune correlate samples from all efficacy trials, as these samples are valuable regardless of the efficacy result.

A novel viral vector in preclinical development as a TB vaccine candidate utilises CMV (cytomegalovirus) as the vector base [172]. Results from the subcutaneous vaccination of rhesus macaques with rhesus CMV encoding nine different *M. tuberculosis* antigens resulted in an overall reduction of *M. tuberculosis* infection by 68% compared with unvaccinated controls, with 41% negative for any disease [172]. The authors’ conclusion of sterilising immunity was based on an absence of radiological disease and negative bacterial cultures from punch biopsies [172]. CMV is highly capable of inducing T_EM_ cells [242–244], though a neutrophil-specific transcriptional signature was found in vaccinated animals [172], suggesting a role for innate immunity. Further work to understand the protective mechanism and how this approach can be successfully translated to the clinic is underway.

## Conclusion

Whilst the field of TB vaccine development has experienced significant hurdles, it is important to recognise the great progress made of late, both in immunological understanding and in empirical learning from human clinical trials. An immunological understanding of the pathogenesis of *M. tuberculosis*, one of the principal barriers to designing an effective vaccine, has slowly but surely been built up to the increasingly clear picture we have today. The classic dogma of the past, focussed solely on the adaptive response, has evolved into something far more complex, integrating the innate, adaptive and humoral systems. With this greater understanding, a variety of novel vaccine design strategies has been made possible. The recent M72/AS01E result gives renewed cause for optimism in this challenging field. It is critical to maintain the momentum that has been built up over the last two decades so that *M. tuberculosis*, a pathogen that has been with us for 3 million years [245], can finally be consigned to the same fate as smallpox.
